# Supplementary material for: The OPRM1 gene and interactions with the 5-HT1a gene regulate conditioned pain modulation in fibromyalgia patients and healthy controls
Source: PLoS One. 2022 Nov 7;17(11):e0277427. doi: 10.1371/journal.pone.0277427 (PMC9639841; doi:10.1371/journal.pone.0277427)
Supplement: Attachment — Submitted filename: Responsetoreviewers.plosOne.docx [file pone.0277427.s001.docx]

Dear Editor,

We thank the reviewers for their suggestions. Please see our point-by-point answers below. We also submit a marked-up copy of our paper with all the changes marked as well as a non-marked up copy.

**Reviewer # 1:** We thank the reviewer for his/her kind words. We are happy that the reviewer found our manuscript interesting, well written and without need for further improvement.

**Reviewer #2:** This is a very interesting study with potentially important implications. The experimental design is excellent and well-described. The statistical analyses are appropriate and considered the nature of repeated testing and potential pain ceiling effects for those participants who had to stop the procedure early. The hypotheses are grounded in the existing literature. Despite these strengths, there appears to be a very significant issue relating to sample size. Because of the nature of the genotype analyses, we would expect that there would be very small numbers of participants in the OPRM1 G-carrier and 5-HT1a CC-genotype groups. Unfortunately, the authors do not present these numbers but it seems likely that there would be fewer than 10 subjects and therefore far fewer than would be ideal to evaluate the effects of interest. This is unfortunate particularly because the manuscript is framed around genotype interactions. After evaluating the study plan for the parent study, it is does not appear that additional participants are likely to be recruited, allowing for appropriate statistical inference. Therefore, this material is better suited as a very exploratory analysis with much less definitive conclusions.

**Answer:**

We thank the reviewer for positive comments of an adequate statistical method and a grounded hypothesis raising this question.

We also thank the reviewer for the relevant comment on sample size. We present the sample sizes with corresponding genotype in Table 2, however they are presented in groups of FM and HC. For our main analyses the groups are pooled, and the exact sample size for the genotypes the reviewer mentions are OPRM1 G-carrier N=28 and for 5-HT1a CC- genotype N=26, i.e. not fewer than 10 individuals. To clarify this in the manuscript, we have added columns to Table 2 for the sample size of the whole group. We believe our sample size is statistically sufficient for making inferences. Yet, we fully agree with the reviewer that the study findings would be more robust with more individuals recruited, however, we are unable to recruit more participants. Regarding the analysis where FM and HC are analyzed separately, the reviewer is correct in that sample size is low and should be inferred as exploratory. We have added a comment in the discussion that this specific analysis is exploratory and needs to be replicated in a larger cohort. Again, we thank for the comment as it has improved our manuscript substantially.

We hope that we have correctly understood the reviewer’s question and provided a satisfactory answer.

Kind regards,

Jeanette Tour, MD, corresponding author
